# Evaluation of a patient-dedicated blood gas analyser

**DOI:** 10.1186/cc14341

**Published:** 2015-03-16

**Authors:** JJ Fox, TH Clutton-Brock

**Affiliations:** 1 Sphere Medical, Cambridge, UK; 2 University of Birmingham, UK

## Introduction

Patient-dedicated arterial blood analysers offer the potential to allow close monitoring of critically ill patients without leaving the patient's bedside or net loss of blood for the patient. A new patient-attached blood gas analyser was evaluated in a clinical environment to compare the results against the reference bench-top analyser.

## Methods

Twenty ICU patients with a range of clinical conditions, including trauma, head injury, post-surgical recovery and sepsis, were included in the study. The new analyser was operated by clinical staff at the Queen Elizabeth Hospital, Birmingham, who each underwent a 90-minute training programme. Patients were monitored using the patient-dedicated analyser (Proxima; Sphere Medical) for up to 3 days. Each time a sample was tested on the patient-dedicated analyser, a concurrent sample was drawn and tested on the hospital's bench-top analyser (Cobas b221; Roche Diagnostics). Samples were assessed using methods described by Bland and Altman for repeated measures. Performance of the novel device was analysed by calculating bias as the mean difference between the new analyser and the comparator device, imprecision as ±1 standard deviation (SD) from the mean and limits of agreement as ±1.96 SD from the mean. Percentage values for bias and precision were calculated from analysis of the percentage difference between the two methods of analysis. Intra-device imprecision for the haematocrit (Hct) sensor was calculated using a pooled variance calculation.

## Results

Over 275 paired samples were analysed over the following concentration ranges: pH 7.249 to 7.545; pCO_2_ 3.32 to 11.01 kPa; pO_2 _5.59 to 21.80 kPa; Hct 23.6 to 41.4%; K+ 3.3 to 4.79 mM. The imprecision for each sensor was calculated to be: pH -0.00 ± 0.03; pCO_2 _-0.48 ± 0.34 kPa; pO_2 _-0.85 ± 0.96 kPa; Hct -4.49 ± 3.42%; K+ 0.08 ± 0.15 mM, respectively. The data collected on the new analyser for Hct showed relative imprecision of 3.42%. The pooled SD was calculated to be 1.21% and the mean SD of each of the novel devices used was 1.14%. This indicates that scatter observed on the Hct sensor was largely due to inter-device rather than intra-device factors.

## Conclusion

The patient-dedicated blood gas analysers demonstrated excellent agreement with the bench-top analyser for pH, pCO_2_, pO_2_ and K+, while Hct provides a reasonable trend monitor with variable bias. Proxima is well suited to enable staff to more closely manage unstable and critically ill patients. This device may be of significant benefit to patients at risk of iatrogenic anaemia or those being treated in side rooms/isolation rooms.

